# Therapeutic alternatives for supporting GPs to deprescribe opioids: a cross-sectional survey

**DOI:** 10.3399/bjgpopen18X101609

**Published:** 2018-11-14

**Authors:** Ruth A White, Chris Hayes, Allison W Boyes, Simon Chiu, Christine L Paul

**Affiliations:** 1 Pain Physiotherapist, School of Medicine and Public Health, University of Newcastle, Newcastle, Australia; 2 Pain Physiotherapist, Hunter Integrated Pain Service, Hunter New England Health, Newcastle, Australia; 3 Director, Hunter Integrated Pain Service, Hunter New England Health, Newcastle, Australia; 4 NHMRC Early Career Fellow, Faculty of Health & Medicine, School of Medicine and Public Health, University of Newcastle, Newcastle, Australia; 5 NHMRC Early Career Fellow, Hunter Medical Research Institute, Newcastle, Australia; 6 Statistician, Hunter Medical Research Institute, Newcastle, Australia; 7 Associate Dean, School of Medicine and Public Health, University of Newcastle, Newcastle, Australia; 8 Co-Deputy Director, Hunter Cancer Research Alliance, Hunter Medical Research Institute, Newcastle, Australia

## Abstract

**Background:**

GPs are central to opioid strategy in chronic non-cancer pain (CNCP). Lack of treatment alternatives and providers are common reasons cited for not deprescribing opioids. There are limited data about availability of multidisciplinary healthcare providers (MHCPs), such as psychologists, physiotherapists, or dietitians, who can provide broader treatments.

**Aim:**

To explore availability of MHCPs, and the association with GP opioid deprescribing and transition to therapeutic alternatives for CNCP.

**Design & setting:**

Cross-sectional survey of all practising GPs (*N* = 1480) in one mixed urban and regional Australian primary health network.

**Method:**

A self-report mailed questionnaire assessed the availability of MHCPs and management of their most recent patient on long-term opioids for CNCP.

**Results:**

Six hundred and eighty-one (46%) valid responses were received. Most GPs (71%) had access to a pain specialist and MHCPs within 50 km. GPs’ previous referral for specialist support was significantly associated with access to a greater number of MHCPs (*P* = 0.001). Employment of a nurse increased the rate ratio of available MHCPs by 12.5% (incidence rate ratio [IRR] 1.125, 95% confidence interval [CI] = 1.001 to 1.264). Only one-third (32%) of GPs reported willingness to deprescribe and shift to broader CNCP treatments. Availability of MHCPs was not significantly associated with deprescribing decisions.

**Conclusion:**

Lack of geographical access to known MHCPs does not appear to be a major barrier to opioid deprescribing and shifting toward non-pharmacological treatments for CNCP. Considerable opportunity remains to encourage GPs' decision to deprescribe, with employment of a practice nurse appearing to play a role.

## How this fits in

Chronic pain, when coupled with low socioeconomic factors and high opioid utilisation, presents a difficult conundrum in the general practice setting. Despite evidence of an unfavourable balance of efficacy and harm with long-term opioids, little is known about the unique clinical challenge of opioid deprescribing in primary care. Close engagement with MHCPs capable of delivering behavioural treatments is considered best practice. This cross-sectional study shows that while MHCPs may be available, they are not currently being used to their full potential in many clinical encounters.

## Introduction

Across Australian and British general practice, the reported prevalence of people experiencing CNCP is 19% and 33–50% respectively, representing a substantial health burden.^1,2^

Developed countries have focused on pharmacological treatments and prescribing rates have subsequently increased.^3^ Although opioid treatment is established as safe and effective for acute and cancer pain,^4^ it has been shown to be no better than a placebo in reducing CNCP.^5^ A recent randomised controlled trial (RCT) for chronic back and osteoarthritis-associated pain found that pain intensity at 12 months was worse in the opioid group compared to the non-opioid treatment arm.^6^

Australian Pharmaceutical Benefits Scheme data identified that opioid prescribing rates exhibit substantial geographic variation, resulting in the proposition that '... differences in access to alternative pain management options may be a factor'.^7^

In the US, the Troup study^8^ identified 90 days as important when shifting towards potentially more effective treatments in primary care and reducing opioid reliance.^9–13^ Large US healthcare groups have been working toward optimal opioid stewardship, with one group achieving a 30% reduction in high dose prescriptions by utilising MHCPs to provide exercise and cognitive behavioural therapy.^14,15^ British guidance recognises the role of the patient and trained non-specialist MHCPs to implement behavioural interventions.^16^ In Australia, GPs are able to create various primary care teams using government-funded general practice management plans (GPMPs). This funding supports consultations with a range of MHCPs including psychologists, physiotherapists, pharmacists, occupational therapists, exercise physiologists, social workers, and dietitians. Given that GPs can create various combinations of providers, it is important to examine the availability of such teams.

This study aimed to identify each of the following among a large mixed urban, and regional sample of Australian GPs: 

1. the proportion of GPs with access to various MHCPs required to potentially implement broader treatments for people experiencing CNCP;
2. whether demographic (sex, year of graduation, qualifications, interest in CNCP, or past referral to a tertiary pain service) and practice characteristics (number of GPs in practice, whether practice nurse is employed, percentage of caseload with CNCP, and co-location of MHCP services) are associated with access to MHCPs for treating CNCP; and
3. whether greater access to MHCPs is associated with increased likelihood of initiating opioid deprescribing for their most recent CNCP utilising long-term opioids.

## Method

### Study design and population

A cross-sectional survey of GPs in one Australian primary health network —﻿ Hunter New England Central Coast Primary Health Network (HNECCPHN) — was conducted between February and April 2016. The HNECCPHN spans a socioeconomically disadvantaged area with 30% of households experiencing rental stress (compared to 25% nationally), 5% of people receiving unemployment benefits long-term (4% nationally), and 4.2% of people identifying as Aboriginal and Torres Strait Islander (2.5% nationally).^17^

Participants were GPs listed on the HNECCPHN register as at February 2016. GPs with incorrect addresses were excluded.

### Procedure

**Figure 1.**
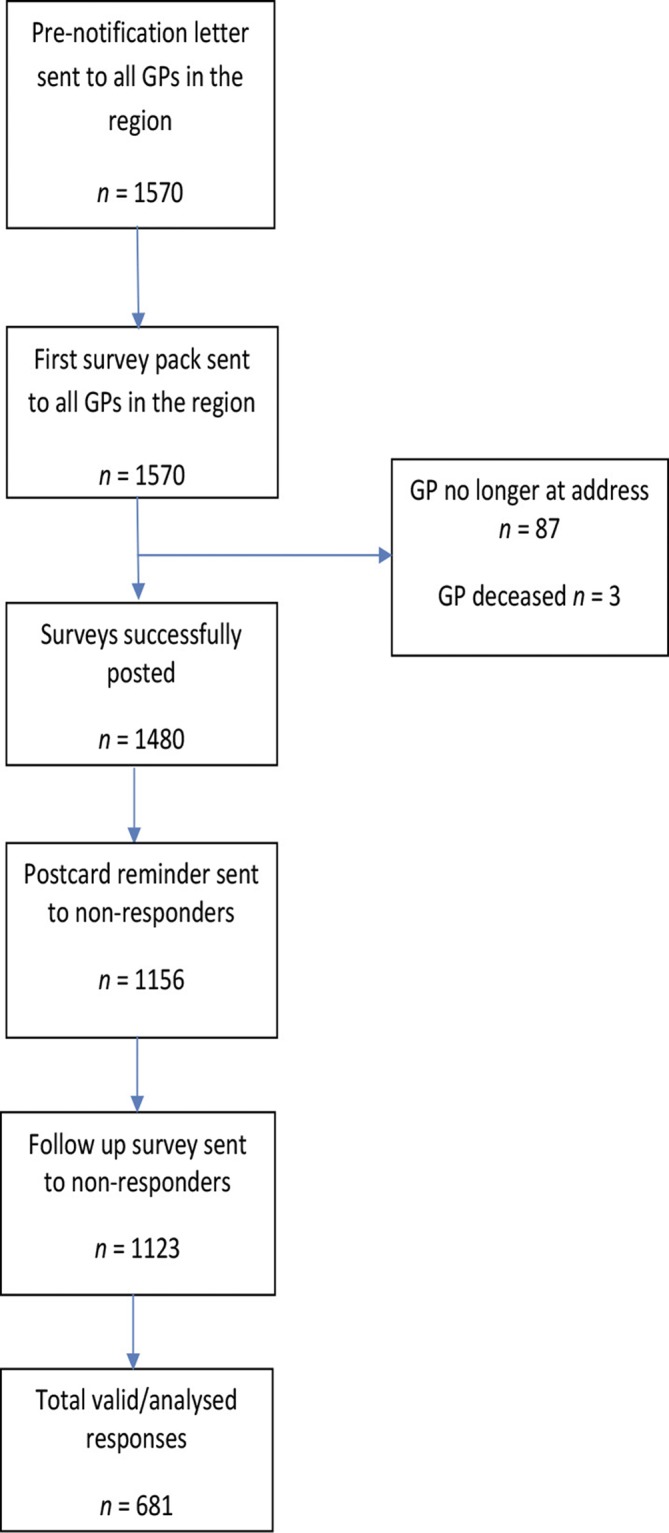
Survey recruitment flowchart.

A multistep recruitment procedure was used (Figure 1).^18^ A personalised pre-notification letter was mailed in February 2016 to introduce the survey and summarise current best practice at the same time as an HNECCPHN newsletter item. The first survey pack (*n* = 1570), mailed in March 2016, was personally addressed to each GP and contained a copy of the questionnaire, a personalised cover letter, details of the chance to win a sports watch valued at AU$500, and a reply paid envelope. The University of Newcastle was identified as the sender and the paper survey had a responder-friendly design.^19–22^ Returns to sender were tracked.^23–26^ Two weeks after the initial mail-out, a professionally designed postcard reminder was mailed to non-responders.^18,27^ A final mailing of the survey package was sent to non-responders 4 weeks after the pre-notification letter.

### Study measure

A 24-item study-specific questionnaire was developed by the authors using current best evidence and expert clinician input. The questionnaire was pilot tested with three GP prescribers to ensure accuracy, face validity, and completion within 10 minutes. Only items relevant to the study aims are described here.

### Sample size

A sample of 500 GPs was sufficient to estimate ±4% for the variables of interest with 80% power.

### Variables of interest

#### Demographic and practice variables

The survey items were: sex (male, female); year of graduation; qualifications higher than foundation degree (yes, no); special interest working with CNCP (yes, no); previous referral of patient to tertiary pain service (Hunter Integrated Pain Service (HIPS) or Tamworth Integrated Pain Service (TIPS), HIPS/TIPS and other, other, never); full-time equivalent staff (1, 2–4, 5–10, >10); practice nurse (yes, no); current clinical caseload for CNCP (0, <5%, 5–10%, >10%).

The survey asked GPs to indicate which MHCPs were available within 50 km of their main practice to form a potential team for care. Response options were; pain specialist, pharmacist, physical therapist, occupational therapist, social worker, exercise physiologist, dietitian, none, other. Responders were asked if any of the MHCPs were co-located (yes, no, partially).

#### Utilisation of MHCPs in most recent patient with CNCP who had been utilising opioids for ≥90 days

The item asked which approach was taken with the most recent CNCP patient taking prescribed opioids for ≥90 days. Response options were derived from review of local clinical guidelines which promoted 90 days as the time point to consider deprescribing:^28^ not applicable, I do not prescribe opioids for this patient group; continued opioid prescription with dose adjustment to maintain pain relief; rotated to another opioid to maintain pain relief and contain dose escalation; initiated gradual opioid weaning to cessation programme; initiated broader primary team care without weaning; or initiated switch to broader team care with specific therapeutic goal to wean opioids to cessation; other. Responses were dichotomised as either unlikely to initiate weaning (for example, ‘continued opioid prescription with dose adjustment to maintain pain relief’; ‘rotated to another opioid to maintain pain relief and contain dose escalation’ or ‘initiated broader primary team care without weaning’) or likely to initiate weaning (for example, ‘not applicable, I do not prescribe opioids for this patient group’; ‘initiated gradual opioid weaning to cessation program’ or ‘initiated broader primary team care with specific therapeutic goal to wean opioids to cessation’).

### Statistical analysis

Data were analysed using STATA (version 14). Percentages with 95% CI are reported for categorical outcomes. Additional outcome variables were created based on a priori hypotheses:

- The total number of available MHCPs by summation of all available MHCPs.
- Whether a GP had a particular combination of available MHCPs: high MHCP access availability (access to a pain specialist), moderate MHCP availability (access to two or more MHCPs, but not a pain specialist), and poor access (no access to a pain specialist and access to one or fewer MHCPs).

Crude and adjusted Poisson regressions were used to examine which sociodemographic factors were associated with a greater total number of MHCPs, high versus moderate/poor access to MHCPs, and likelihood of the GP to wean their most recent CNCP patient off prescribed opioids. The regression analysis of likelihood to wean also included the access to MHCPs variable. The relatively high number of total MHCPs and low variance indicated that the distribution was under dispersed. Robust variance estimators were used to estimate the coefficient standard error to protect against biases. Logistic regression was used to measure associations between access to MHCPs and demographic, practice characteristics, as well as the GPs likelihood to initiate broader care. Each of the covariates were modelled separately, then collectively in an adjusted model. The reference category for the logistic regression was set as ‘poor/moderate access’ to measure the odds of ‘high access’.

## Results

### Sample

Of the 1570 mailed postal questionnaires, 1480 were delivered and 681 were completed. Of the 90 undeliverable surveys, three were due to ‘GP deceased’ and 87 due to ‘GP no longer working at the practice or retired'. The total valid adjusted response rate was 46%. There were no significant differences between responders and non-responders in regard to their sex.

Demographic characteristics of the sample are shown in Table 1. Female GPs accounted for 43% of responders which is consistent with national figures.^29^ Most practices (*n* = 627, 92%) employed a practice nurse, which is higher than a 2012 finding of 63%.^23^

**Table 1.** Demographics of Hunter New England Central Coast Primary Health Network GPs

		*n* = 681	%	95% CIs
**Individual GP characteristics**				
Sex				
Male		390	57.3	54.6 to 61.0
Female		290	42.6	39.0 to 46.4
**Year of graduation**				
Before 1995		396	58.1	56.9 to 64.4.
1995–2005		183	26.8	24.7 to 31.7
2006–2010		60	8.8	7.2 to 11.7
2011–2014		13	1.9	1.2 to 3.4
**Higher qualification**				
Yes		472	69.3	66.7 to 73.6
No		200	29.3	26.4 to 33.3
**Special interest in pain**				
Yes		174	25.6	22.7 to 29.3
No		499	73.3	70.7 to 77.3
**Past referral to a pain clinic?**				
HIPS/TIPS		346	50.8	47.9 to 55.5
HIPS/TIPS + other		236	34.7	31.7 to 39.0
Other		69	10.1	8.2 to 12.9
Never		18	2.6	1.7 to 4.2
**Current caseload of CNCP, %**				
0		4	0.6	0.2 to 1.6
<5		200	29.4	26.5 to 33.5
5–10		339	49.8	46.9 to 54.5
>10		126	18.5	16.0 to 22.0
**Practice characteristics**				
FTE GPs at practice				
1		64	9.4	7.5 to 12.0
2–4		255	37.4	34.3 to 41.6
5–10		297	43.6	40.4 to 47.9
>11		57	8.4	6.6 to 10.8
**Practice nurse**				
Yes		627	92.1	90.8 to 94.7
No		47	6.9	5.3 to 9.2

CI = confidence intervals. CNCP = chronic non-cancer pain. FTE = full-time equivalent. HIPS = Hunter Integrated Pain Service. TIPS = Tamworth Integrated Pain Service. Totals may not add to 681 (or 100%) due to missing data.

Compared to national data from Bettering the Evaluation and Care of Health (BEACH), which estimated 15% (95% CI = 14 to 17) of patients attending general practice experience CNCP,^1 ^19% (95% CI = 16 to 22) of this sample indicated a similar caseload.

### GP access to MHCPs

**Figure 2.**
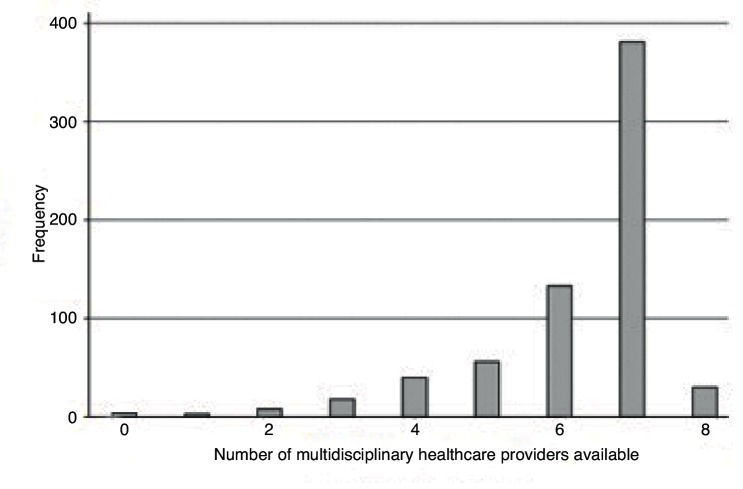
Histogram showing total number of MHCPs available to GP

Figure 2 shows the distribution of the total number of available MHCPs for each responder. The majority of GPs reported access to seven MHCPs (mean 6.27, standard deviation 1.32).

Availability of MHCPs is reported in Table 2. Access to a physical therapist was the most commonly selected MHCP (*n* = 663, 97%). The subgroup combination of physical therapist, pharmacist, and dietitian was available to most GPs (*n* = 620, 91%).

**Table 2.** Availability of resources within 50 km of main practice (for a GPMP/TCA)

	*n* = 681	%	95% CIs
**Access to multidisciplinary resources**			
Pain specialist			
No	157	23.1	20.3 to 26.7
Yes	516	75.8	73.3 to 79.7
Pharmacist			
No	31	4.6	3.3 to 6.5
Yes	642	94.3	93.5 to 96.7
Physical therapist			
No	10	1.5	0.8 to 2.7
Yes	663	97.4	97.3 to 99.2
Occupational therapist			
No	87	12.8	10.6 to 15.7
Yes	586	86.0	84.3 to 89.4
Exercise physiologist			
No	69	10.1	8.2 to 12.8
Yes	604	88.7	87.2 to 91.8
Dietitian			
No	32	4.7	3.4 to 6.7
Yes	641	94.1	93.3 to 96.6
None			
No	666	97.8	97.8 to 99.5
Yes	7	1.0	0.5 to 2.2
**Access to combinations**			
Combination PS/PH/DT/PT			
No	188	27.6	24.7 to 31.5
Yes	485	71.2	68.5 to 75.3
Combination OT/EP			
No	111	16.3	13.9 to 19.5
Yes	562	82.5	80.5 to 86.1
Combination PS/SW			
No	257	37.7	34.6 to 41.9
Yes	416	61.1	58.1 to 65.4
Combination DT/PT			
No	36	5.3	3.9 to 7.3
Yes	637	93.5	92.7 to 96.1
Combination PT/PH			
No	34	5.0	3.6 to 7.0
Yes	639	93.8	93.0 to 96.4
Missing data	8	1.2	
Combination PT/PH/DT			
No	53	7.8	6.1 to 10.2
Yes	620	91.0	89.8 to 93.9
Combination PS/PT/SW			
No	257	37.7	34.6 to 41.9
Yes	416	61.1	58.1 to 65.4
All providers			
No	275	40.4	37.2 to 44.6
Yes	398	58.4	55.4 to 62.8
Co-location of providers			
All selected	19	2.8	1.8 to 4.4
Some selected	253	37.2	344 to 41.8
None selected	393	57.7	55.3 to 62.8

DT = dietitian. EP = exercise physiologist. GPMP = general practice management plan. OT = occupational therapist. TCA = team care arrangement. PH = pharmacist. PS = pain specialist. PT = physical therapist. SW = social worker. Totals may not add to 681 (or 100%) due to missing data.

### Factors associated with GPs access to MHCPs

The crude modelling suggested that graduating recently, having referred to tertiary pain services in addition to the specified local tertiary pain services, being in a practice with 5–10 GPs rather than a solo practice, and employment of a nurse, were significantly associated with high availability of MHCPs for pain management (*P* = 0.047). After adjusting for all covariates, employment of a nurse and prior referral to ‘other’ tertiary pain services were statistically significantly associated with the number of available MHCPs. The adjusted model, however, is the most important result as it accounts for differences within sample demographics. It is estimated that for a GP whose main practice employed a nurse, there was an increased rate ratio of the number of MHCPs available by 12.5% (IRR 1.125, 95% CI = 1.001 to 1.264). According to the adjusted model, previous referral to both local and ‘other’ tertiary pain services was significantly associated with 7% higher access to MHCPs compared to GPs who had only referred to local tertiary pain services (IRR = 1.07, 95% CI = 1.033 to 1.108, *P* = 001). Further information is available from the authors on request.

### Greater access to MHCPs and deprescribing

Table 3 shows the treatment choices made by GPs for their most recent CNCP patient who had been utilising opioids for ≥90 days.

**Table 3.** Most recent approach with CNCP patient on long-term opioids

	*n* = 681	%	95% CIs
Not applicable, do not prescribe	27	4.0	2.9 to 6.0
Continued opioid prescription	98	14.4	12.6 to 18.2
Rotated to another opioid	38	5.6	4.3 to 8.0
Initiated gradual wean	131	19.2	17.3 to 23.6
Team care, no wean	67	9.8	8.2 to 13.0
Team care with opioid wean	217	31.9	30.0 to 37.3
Other	68	10.0	8.4 to 13.1

CI = confidence intervals. Totals may not add to 681 (or 100%) due to missing data.

The crude models are presented to highlight the effects before and after adjusting for potential confounding factors; however, none of the factors (sociodemographic or access to MHCPs) included in either the crude and adjusted logistic regression models, were significantly associated with reported opioid deprescribing of a CNCP patient (with or without team care) within the past 90 days.

## Discussion

### Summary

This survey was the first of a large sample of urban and regional Australian GPs to examine the geographic availability of known MHCPs required to potentially form a local team to deliver behaviour change treatments for people experiencing CNCP and utilising long-term opioids. The data suggested it is possible to access appropriate MHCPs even in a regional area.

The findings did not support the hypothesis that a lack of availability of known MHCPs is a strong driver of current liberal opioid prescribing. Most GPs had at least moderate access (within 50 km) to form a team of MHCPs capable of providing broader care. It is possible that MHCP availability may influence GP confidence in negotiating treatment alternatives.^30^ However, the view that opioid prescribing is a surrogate for inadequate access to MHCPs was not supported.^31^

Pain services located within Australian tertiary public hospitals actively promote deprescribing of long-term opioids.^32–34^ Responders who had previously referred to these services reported access to a greater number of local MHCPs than those who referred locally only. Willingness to explore MHCP treatment options is considered likely to be a necessary component for improving outcomes for this patient group^1^ and these findings indicate that referral habits are important.

Employment of a practice nurse was positively associated with the number of available MHCPs. It is likely these practitioners are coordinating the shift toward broader care,^35–37^ which is congruent with the literature.

The data failed to show any association between MHCP accessibility and likelihood of opioid deprescribing. Only about one-third took the recommended approach of shifting toward broader treatments plus deprescribing. These findings suggest that Australian GPs are beginning to exercise good stewardship via referrals for specialist support to assist with weaning. Pain specialists are a relatively ‘expensive’ resource, however, allocating more funding for medical specialist input could be helpful if integrated with primary care. The extent of uptake of GPMPs for initiating a rehabilitation approach for CNCP is not known.^38^ Reasons why GPs do not initiate opioid deprescribing are not well known, however a recent study of early-career GPs identified potential barriers, including gaps in undergraduate training.^39^

### Strengths and limitations

The major strength of this study is the large sample size. It is also the first study to provide a profile of resources available to GPs in the region. 

Recall bias by GPs asked about MHCP availability may have limited the accuracy of study findings. However, there is no accurate and accessible database to objectively assess availability of MHCPs. The survey response rate while low, compares favourably to other surveys of GPs.^40–42^ The response rate may result in a lack of precision in the study data. Information on distribution of MHCPs collected in this survey may not be generalisable to other rural and remote areas.^43^ This study's definition of access fails to capture other facets such as affordability and appropriateness, and therefore provides an overestimate of ‘true’ access. Other non-MHCP resource related influences on GPs’ willingness to initiate deprescribing, such as patient pressure and pharmaceutical marketing, were not explored.^44,45^ Asking GPs about their most recent patient has limitations as this patient may not be typical.

### Comparison with existing literature

This is the first study specifically exploring the accessibility of therapeutic alternatives when GPs are considering deprescribing opioids. Existing literature has been growing on the disturbing rise in opioid prescription and clinicians are being urged to consider more cautious prescribing.^9^ A recent observational study using Veterans Health Administration databases from 2010–2016 suggested that long-term opioid prescribing is declining, however the role of therapeutic alternatives was not explored.^46^ The UK-based COPERS trial was a multi-centre, pragmatic trial across 27 general practices aimed at reducing pain-related disability. While the brief intervention did not achieve the desired outcome, it certainly suggested that access to therapeutic alternatives could improve the psychological well-being of people with CNCP.^47^

### Implications for research and practice

The results of this survey among Australian GPs suggests that availability of known MHCPs is not likely to be a major barrier in shifting towards non-pharmacological treatment for CNCP, at least in urban and regional primary care settings. Sociodemographic and practice characteristics provide very little further explanation of GPs’ decision to continue rather than wean opioids. Globally, there is a need to identify and test whether standard practice can be shifted towards treatments which promote behaviour change. Such care, delivered by experienced and appropriately trained MHCPs, may be a viable non-pharmacological alternative for people with CNCP.
